# Withering and Fermentation Affect the Transformation and Accumulation of Key Metabolites in Rougui (*Camellia sinensis*) for the Formation of Special Taste Characteristics

**DOI:** 10.3390/foods13233955

**Published:** 2024-12-08

**Authors:** Jianghua Ye, Yangxin Luo, Yulin Wang, Qi Zhang, Shuqi Zhang, Junbin Gu, Yankun Liao, Tingting Wang, Xiaoli Jia, Haibin Wang

**Affiliations:** 1 College of Tea and Food, Wuyi University, Wuyishan 354300, China; jhye1998@126.com (J.Y.);; 2 College of Life Science, Longyan University, Longyan 364012, China

**Keywords:** Wuyi rock tea, withering, fermentation, key metabolites, taste characteristics

## Abstract

During the production of Wuyi rock tea, withering and fermentation play a crucial role in the primary processing of the tea, greatly influencing the development of its distinct taste characteristics. In this study, Rougui (*Camellia sinensis*) was selected as the research object to investigate the effects of withering and fermentation on metabolites and taste characteristics in tea leaves. The findings revealed that a total of 1249 metabolites were detected in Rougui leaves at various processing stages, of which only 40 key metabolites were significantly altered. The process of withering and fermentation is crucial to increase the content of organic acids, plumerane, alkaloids, nucleotides and derivatives, amino acids and derivatives, and free fatty acids in the leaves of Rougui and to decrease the content of saccharides, phenolic acids, flavonols, flavones, and flavanols, which in turn enhances the mellowness, fresh and brisk taste, and aroma of tea and attenuates the saccharides, bitterness, and astringency. Withering and fermentation had the greatest effect on the bitterness and astringency of Rougui taste characteristics, followed by mellowness. It can be seen that withering and fermentation were extremely important for the development of Rougui’s special taste characteristics. The present study provides important support for optimizing Rougui processing and the formation of its special taste characteristics.

## 1. Introduction

Wuyishan is an influential tea-producing region located at 27°32′36″~27°55′15″ north latitude and 117°24′12″~118°02′50″ east longitude in Fujian Province, China. The unique geological structure of the region, characterized by Danxia geomorphology, is essential for cultivating and producing the renowned Wuyi rock tea, a type of oolong tea with a more intricate process compared to other teas [1]. The primary processing of Wuyi rock tea is divided into two distinctive stages. The first stage involves the fresh leaves’ picking, withering, and fermentation, while the second stage mainly involves the green removing, rolling, and drying of the leaves. During the first stage, the chemical composition of the tea leaves undergoes significant changes under the influence of both enzymatic and non-enzymatic reactions, which contribute to the formation of the unique flavor of Wuyi rock tea [2,3]. In the subsequent second stage, after conducting green removing on the tea leaves, the chemical transformation occurs exclusively under non-enzymatic reactions. Therefore, the primary processing stage is the primary determinant of the chemical composition of Wuyi rock tea, with the first stage being particularly critical as it lays the foundation for the second stage and is a crucial factor in the formation of the distinct flavor profile [4,5,6,7]. This identifies the significant role that the first stage of the primary processing plays in the production of Wuyi rock tea and the crucial importance of understanding the metabolite change of each node in the production process.

Wuyi rock tea’s primary processing is mainly divided into three steps: picking fresh leaves, withering, and fermentation. Tea leaves are carefully picked and then undergo withering, the purpose of which is to induce a moderate loss of water and soften the leaves, and this process encourages the transformation and accumulation of substances within the leaves [8]. For example, Zhou et al. [9] discovered that withering could reduce the total catechin and starch content while increasing the theaflavin, γ-aminobutyric acid, maltose, and soluble sugar content, thereby enhancing the mellow, umami, and sweet taste of white tea. Furthermore, Fang et al. [10] analyzed the effect of withering on black tea metabolites, revealing that withering encouraged the production of alcohols, aldehydes, phenols, hydrocarbons, and halogenated hydrocarbons. Similarly, Shan et al. [11] found that most free amino acids and catechins increased in content after the withering of Longjing green tea, while the content of organic acids, phenolic acids, and their derivatives decreased. This indicates that during withering, the substances inside the tea leaves have already started undergoing transformation and accumulation, and this transformation and accumulation of substances was differential among different types of tea leaves. Once withering is complete, tea leaves are ready for fermentation. During this stage, the enzyme activity within the leaves rapidly increases, intensifying the degree of substance transformation and rapidly raising the content of the total substances [12]. For example, Salman et al. [13] found that fermentation significantly affects the substance transformation of tea, with the higher the degree of fermentation, the higher the content of theaflavins and the lower the content of catechins and total phenols. Wang et al. [14] found that fermentation promotes the oxidative degradation of fatty acids and carotenoids in black tea, thus enhancing the formation and accumulation of terpenoids, ketones, and aldehydes. It can be seen that during the primary processing of tea, withering and fermentation have an important impact on the transformation and accumulation of substances in tea, and for different types of tea, the effect of withering and fermentation on the transformation of substances is obviously different, which leads to different types of tea with different flavors. For instance, Jia et al. [15] concluded that withering is beneficial for enhancing the mellowness, freshness, and brisk taste of oolong tea, and there are 47 characteristic metabolites determining the changes in the taste characteristics, mainly nucleotides and derivatives, terpenoids, and organic acids. Li et al. [16] found that only 71 key metabolites, which are categorized as catechins, flavonoids, phenolic acids, and terpenoids, significantly changed in Fu brick tea after withering and fermentation. Secondly, withering and fermentation reduced the bitterness, astringency, and acidity of Fu brick tea but increased the mellowness of the tea. Cheng et al. [17] found that withering and fermentation are conducive to reducing the bitterness and astringency of Qingzhuan tea and improving the woodiness and mellowness of the tea, and that there were 102 key metabolites determining this change in taste characteristics. It can be seen that the formation of tea taste characteristics was closely linked to changes in key metabolites during processing, and the analysis and acquisition of key metabolites with significant changes during tea processing were of significance for enhancing tea processing and shaping tea flavor.

Rougui (*Camellia sinensis*) is the primary cultivated variety of tea trees in Wuyishan, a representative location of Wuyi rock tea. However, the key metabolites that significantly change after withering and fermentation in Rougui and their effects on tea taste characteristics are still unclear. In-depth investigation into the effect of withering and fermentation on the transformation and accumulation of Rougui metabolites, as well as obtaining the key metabolites and analyzing their effects on the taste characteristics of the tea, are of great significance for optimizing the processing technology of Rougui tea and improving its taste characteristics. Therefore, in the present study, Rougui was chosen as the subject of research. Initially, the tea leaves were processed in accordance with the traditional Wuyi rock tea production method, and fresh leaves, withered leaves, and leaves at different fermentation degrees were collected to assess the effects of different processing processes on the metabolite content of leaves. Meanwhile, key metabolites were screened to obtain significant changes in the different processing nodes and then analyzed for their taste characteristics. The present study analyzed the effect of withering and fermentation on the taste characteristics of Rougui, which is crucial in Rougui processing from a practical production standpoint, providing valuable information for refining Rougui processing and producing superior-quality Rougui tea.

## 2. Materials and Methods

### 2.1. Field Experiment and Sample Collection

**Figure 1:**
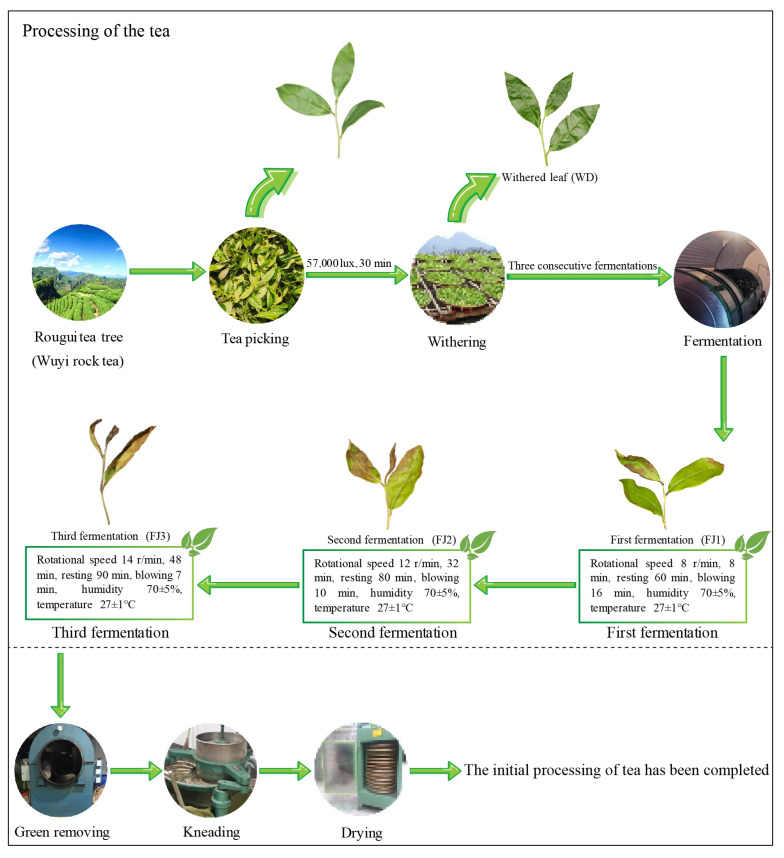
Primary processing of Wuyi rock tea and distribution of sampling points.

Located in Fujian Province, Wuyishan City, China, is an important tea-producing region in the subtropical zone. It experiences an average annual temperature of 12~13 °C, with an annual precipitation of more than 2000 mm and a relative humidity of 85%. Wuyi rock tea is a famous traditional Chinese tea because tea trees grow in the cracks of rock, and the tea therefore has a special rock rhyme quality. Rougui tea tree is a highly recognized cultivar native to the renowned Wuyishan region of Fujian Province. This cultivar was formally recognized as a superior provincial variety by the Crop Variety Validation Committee of Fujian Province in 1985. In this study, fresh leaves of the Rougui tea tree (three leaves and one core) were collected, and the tea leaves were primary-processed according to the traditional production mode of Wuyi rock tea, including fresh leaf picking, withering, fermentation, green removing, kneading, and drying [18]. This study focuses on metabolite changes in Rougui leaves at various processing nodes during the first stage of primary processing and their effects on taste characteristics. Therefore, fresh leaves (FL), withered leaves (WD), and leaves after the 1st, 2nd, and 3rd fermentations (FJ1, FJ2, FJ3) of Rougui processing were collected in this study (Figure 1). Meanwhile, the Rougui leaves collected were extracted, enriched, and analyzed for metabolites using ultra-performance liquid chromatography/tandem mass spectrometry (UPLC-MS/MS) with three replicates per sample.

### 2.2. Metabolite Extraction from Tea Leaves

The tea samples collected were first freeze-dried through a vacuum freeze-dryer (Scientz-100F, Hangzhou, China). The dried samples were ground into powder and subsequently used for the metabolite extraction as described by Zhou et al. [19]. In brief, 60 mg of tea powder was added to 1.5 mL of methanol (70%) and stirred with rapid vortexing and shaking for 45 s. For this process, in a vortex mixer, the prepared sample was agitated for 45 s at 30 min intervals, with the overall number of vortex agitation cycles being 6. Subsequently, the sample was centrifuged in a centrifuge at a speed of 12,000 revolutions per minute for 3 min, and the supernatant was isolated utilizing an ANPEL SCAA-104 0.22 μm filter membrane (Shanghai, China). This isolated supernatant was then utilized for the UPLC-MS/MS (UPLC, Shimadzu’s Nexera X2, Kyoto, Japan; MS/MS, Applied Biosystems 4500 QTRAP, MA, USA) analysis. Three independent replicates were set up for each sample.

### 2.3. Qualitative and Quantitative Analysis of Metabolites in Tea

Metabolites were determined using UPLC-MS/MS, in which the UPLC was Shimadzu’s Nexera X2 (Kyoto, Japan), the column was the Agilent SB-C18 (1.8 µm, 2.1 mm × 100 mm, CA, USA), and the MS/MS was the Applied Biosystems 4500 QTRAP (MA, USA). Metabolite analysis was conducted utilizing ultra-performance liquid chromatography (UPLC) with a gradient elution mode. The mobile phase consisted of ultrapure water with 0.1% formic acid serving as mobile phase A and acetonitrile with 0.1% formic acid functioning as mobile phase B. The UPLC run was carefully designed to achieve a ratio of mobile phase A initially at 95% at 0.00 min, subsequently gradually decreasing to 5% over a period of within 9.00 min and being maintained for an additional 1 min. Subsequently, the proportion of phase A was increased to 95% within 1.1 min and maintained for 2.9 min. The injection volume of the sample during the UPLC assay was kept at 4 μL, the flow rate was set at 0.35 mL/min, and the column temperature was maintained at 40 °C. The MS/MS conditions for metabolite identification were set as follows: the source temperature was set to 550 °C; the ion spray voltage was adjusted to 5500 V for the positive ion mode and −4500 V for the negative ion mode; the collision-activated dissociation was programmed to high; the compositions of the ion sources gas I, gas II, and the curtain gas were adjusted to 50, 60, and 25 psi, respectively; and the spectral scanning was performed by employing a triple series quadrupole mass spectrometer configured for the multiple reaction monitoring mode. Prior to scanning, the instrument was adjusted to an intermediate level of collision gas intensity. It then underwent a series of tuning and mass calibration procedures using 10 and 100 μmol/L of polypropylene glycol solutions as reference substances. The principle applied was to determine the optimal declustering potential and the collision energy of each multiple reaction monitoring ion in order to track specific multiple reaction monitoring ion pairs of eluted metabolites across all subsequent time periods. The characterization of the metabolites was facilitated by the application of advanced mass spectrometry data analysis methods utilizing Analyst (1.6.3) software to obtain retention times and ion current intensities for each metabolite. This approach was further refined by facilitating direct comparisons with NIST20 mass spectrometry database standards. Following the characterization of the metabolites, the characteristic ions and their signal intensities for each metabolite were determined using a triple quadrupole mass spectrometer. Subsequently, the chromatographic peaks corresponding to each metabolite were integrated and corrected using the software package MultiQuant (version 3.0). The peak areas derived from the chromatographic peaks were utilized as a surrogate measure of the metabolite content [20].

### 2.4. Statistical Analysis

In this study, the raw data of the metabolites were obtained, and, firstly, Excel 2020 was used for the preliminary statistical analysis, including the calculation of the mean value of metabolite content and its classification. Differences in metabolite content across different processing nodes were analyzed using variance analysis and paired Student’s t-tests, with *p* < 0.05 being considered statistically significant. After data analysis, graphs were produced uniformly using Rstudio software (v 4.2.3) and different R packages [21]. The R package used for the multi-group box plots was gghalves 0.1.4; for the heat maps, it was pheatmap 1.0.12; for the principal component analysis, it was ggbiplot 0.55; for the Kruskal–Wallis test and LDA analysis, it was microeco; and for the multiple volcano plots, it was ggplot2 and ggnewscale. The R package used for technique for order preference by similarity to ideal solution (TOPSIS) was dplyr 1.1.4, and the R package used for the curvilinear plot was ggplot2.

## 3. Results and Discussion

### 3.1. Effects of Withering and Fermentation on Metabolites in Tea Leaves

**Figure 2:**
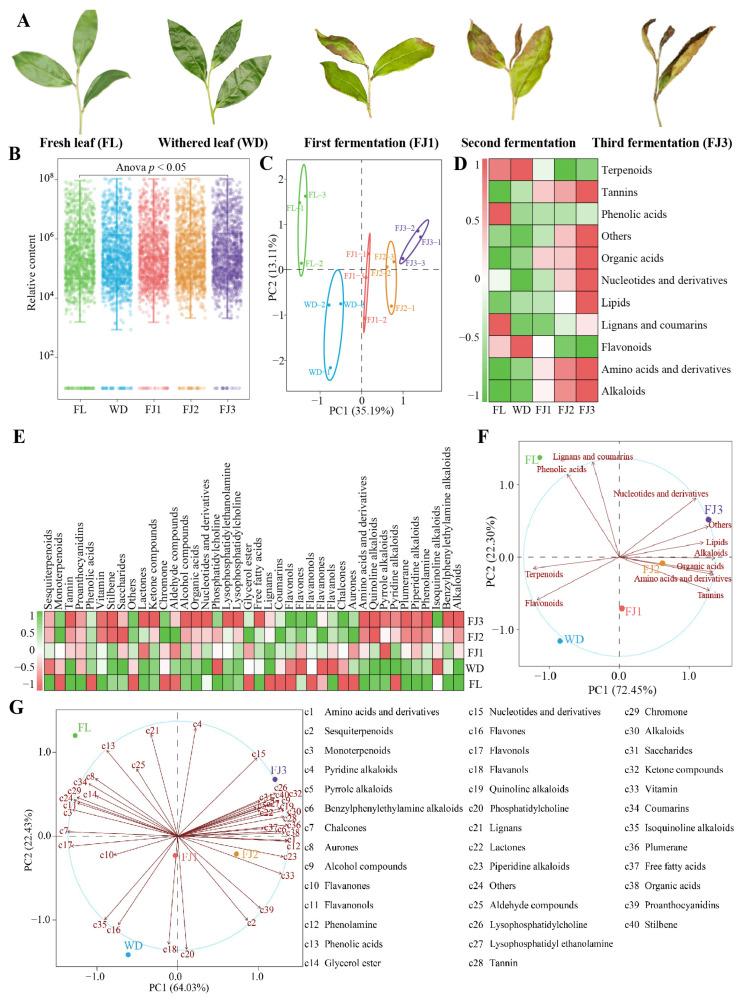
Metabolomics analysis of tea leaves across different processing nodes. Note: FL: fresh leaves; WD: withered leaves; FJ1: first fermentation; FJ2: second fermentation; FJ3: third fermentation; (**A**): tea leaf morphology across different processing nodes; (**B**): metabolite contents in tea leaves across different processing nodes; (**C**): principal component analysis of metabolite contents in tea leaves across different processing nodes; (**D**): heat map of metabolite contents of primary classification in tea leaves at different processing nodes; (**E**): heat map of metabolite contents of secondary classification in tea leaves across different processing nodes; (**F**): principal component analysis of metabolite contents of primary classification in tea leaves across different processing nodes; (**G**): principal component analysis of metabolite contents of secondary classification in tea leaves across different processing nodes.

During processing, substances in tea leaves are transformed and accumulated, and the composition and content of different substances change, which has an important role in the special flavor of tea [2,22]. Wang et al. [23] found that the metabolites in green tea leaves changed significantly during processing, and 527 key metabolites were obtained, which could be categorized into 11 groups. Chen et al. [24] detected the metabolites of oolong tea processing and revealed that the key metabolites that underwent significant changes in oolong tea as a result of processing were catechins and their derivatives, flavonol glycosides, and amino acids. In the present study, it was revealed (Figure 2A) that significant morphological changes occurred during Rougui processing from fresh leaves (FL) to withering leaves (WD) to fermented leaves (FJ1 to FJ3), and that such changes may lead to alterations in the transformation and accumulation of metabolites. Therefore, this study deeply analyzed leaf metabolite changes during Rougui processing through the UPLC-MS/MS technique, and the results revealed (Figure 2B, Table S1) that during the processing of Rougui leaves, a total of 1249 metabolites were detected at each node; moreover, the total amount of metabolites significantly increased with each processing step. Principal component analysis on leaf metabolite content from different processing nodes showed (Figure 2C) that the two principal components could effectively distinguish the different processing nodes. The contribution of principal component 1 was found to be 35.19%, while the contribution of principal component 2 was 13.11%. This resulted in an overall contribution of 48.30%. It is evident that the metabolite content in the leaves significantly altered during the processing of Rougui.

The present study continued to analyze the classification of metabolites in Rougui from different processing nodes, and the results showed (Figure 2D) that metabolites were characterized based on the first level of classification, where they could be phylogenetically organized into eleven groups. Five of these groups, encompassing amino acids and derivatives, tannins, alkaloids, organic acids, and lipids, exhibited an increasing trend in their content. Conversely, the content of the four other groups, encompassing phenolic acids, nucleotides and derivatives, lignans and coumarins, and others, displayed a biphasic trend, initially decreasing and then increasing. The secondary classification of metabolites demonstrated (Figure 2E) that they could be categorized into 40 groups. Of these, 10 groups displayed a progressive increase, 4 groups exhibited a progressive decrease, 3 groups exhibited a gradual increase followed by a subsequent decrease, 10 groups demonstrated a gradual decrease followed by a subsequent increase, and 13 groups exhibited fluctuating changes as the tea processing process was executed. The results of the principal component analysis after the first-level categorization of metabolites from different processing nodes showed (Figure 2F) that the contribution of principal component 1 was 72.45%, in which FL, WD, and FJ1 were located at the negative end of principal component 1, with the main contribution coming from four groups of metabolites, while FJ2 and FJ3 were located at the positive end of principal component 1, with the main contribution coming from seven groups of metabolites. The results of the principal component analysis after the secondary categorization of the metabolites from different processing nodes showed (Figure 2G) that the contribution of principal component 1 amounted to 64.03%, in which FL, WD, and FJ1 were still located at the negative end of principal component 1, with the main contribution coming from 16 groups of metabolites, while FJ2 and FJ3 were located at the positive end of principal component 1, with the main contribution coming from 24 groups of metabolites. It is evident that leaf metabolites significantly altered during the processing of Rougui, and significant differences appeared in their contents.

### 3.2. Screening of Key Differential Metabolites Across Different Processing Nodes

**Figure 3:**
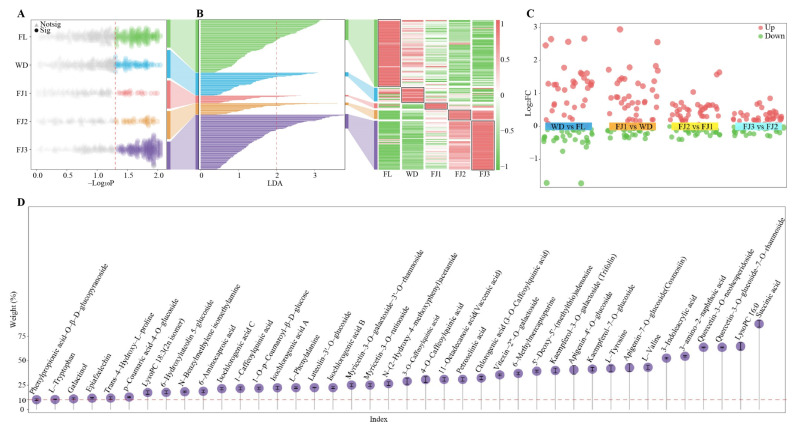
Screening for significantly changed key metabolites in Rougui leaves across different processing nodes. Note: FL: fresh leaves; WD: withered leaves; FJ1: first fermentation; FJ2: second fermentation; FJ3: third fermentation; (**A**): screening for significantly changed metabolites across different processing nodes by Kruskal–Wallis test; (**B**): screening for significantly changed differential metabolites by LDA; (**C**): screening for significantly changed key metabolites using a multiple volcano diagram; (**D**): determination of the weight of the effect of withering and fermentation on key metabolites by TOPSIS.3.3. Taste characteristics of key metabolites.

There is a large variety of metabolites in tea, and processing leads to changes in their types and contents, while a small number of key metabolites are mainly responsible for determining tea taste characteristics [25]. Shi et al. [26] analyzed the effect of processing on green tea metabolites and found that there were up to 1034 metabolites in green tea, while only 146 key metabolites, mainly flavonoids, amino acids, lipids, and phenolic acid metabolites, were significantly changed during processing. Xue et al. [27] revealed that 368 metabolites were obtained during black tea processing, of which only 93 key metabolites were significantly changed, and key metabolites determined tea taste characteristics. It can be seen that screening and obtaining key metabolites that change significantly during tea processing is important for further analyzing the impact of processing on the formation of tea taste characteristics. Accordingly, this study further screened the key metabolites that were significantly changed during the processing of Rougui. In the present study, the metabolite content in Rougui across different processing nodes was analyzed using the Kruskal–Wallis test, and it was found (Figure 3A) that, out of 1249 metabolites, 492 metabolites were significantly changed. Analysis of 492 metabolites using LDA revealed 144 differential metabolites with LDA values greater than two (Figure 3B). Further analysis of 144 differential metabolites that significantly changed across different processing nodes using multiple volcano plots revealed (Figure 3C) that 71 key metabolites that significantly changed were obtained by screening, and 39 of these metabolites exhibited an increase in content and the remaining 32 presented a decrease as processing progressed. The weight of withering and fermentation effects on the content of 71 metabolites was determined using TOPSIS weight analysis, and it was found (Figure 3D) that only 40 of the 71 metabolites had an effect weight greater than 10%. It can be seen that only 40 key metabolites were significantly changed during the withering and fermentation of Rougui, and the content of key metabolites may affect the taste characteristics of tea.

**Figure 4:**
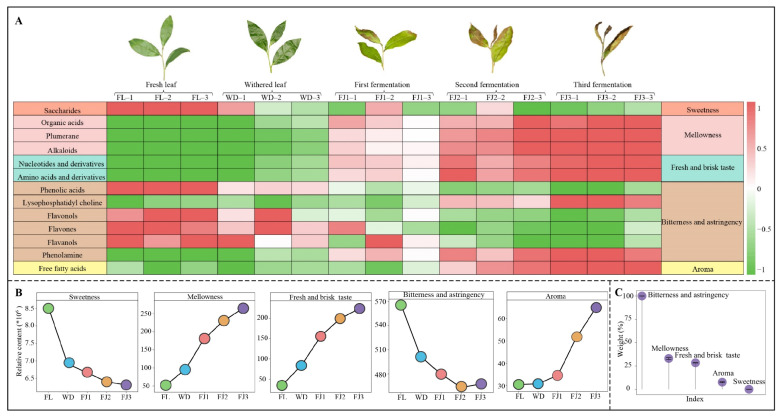
Changes in the content of key metabolites from tea leaves across different processing nodes and their effects on taste characteristics. Note: FL: fresh leaves; WD: withered leaves; FJ1: first fermentation; FJ2: second fermentation; FJ3: third fermentation; (**A**): changes in the content of key metabolites and their taste characteristics at different processing stages; (**B**): trends in the intensity of taste characteristics of tea leaves across different processing nodes; (**C**): TOPSIS determination of the weights of the effects of withering and fermentation on different taste characteristics.

From the above results, 40 key metabolites obtained in this study were further categorized, and it was found (Figure 4A) that the 40 key metabolites were subsequently classified into 13 groups based on the secondary classification. Five of these groups, including saccharides, phenolic acids, flavonols, flavones, and flavanols, exhibited a significant reduction in content upon processing, while eight groups, including organic acids, plumerane, alkaloids, nucleotides and derivatives, amino acids and derivatives, lysophosphatidyl choline, phenolamine, and free fatty acids, demonstrated a significant increase in content. Reportedly, tea leaves exhibit diverse taste characteristics due to the varying types of metabolites. For example, saccharides mainly present sweetness taste characteristics [28]. Organic acids, plumerane, and alkaloids mainly present mellowness taste characteristics [29,30]. Nucleotides and derivatives and amino acids and derivatives mainly present fresh and brisk taste characteristics [31,32]. Phenolic acids, flavonols, flavones, flavanols, phenolamine, and lysine mainly present bitterness and astringency taste characteristics [33,34,35,36]. However, free fatty acids mainly present aroma [37]. It can be seen that, after withering and fermentation, the key metabolites that changed significantly in Rougui mainly exhibited five types of taste characteristics, namely sweetness, mellowness, fresh and brisk taste, bitterness and astringency, and aroma. Further analysis of the trends in the intensity of the five taste characteristics during the processing of Rougui revealed (Figure 4B) that the intensity of the sweetness, bitterness, and astringency of the tea showed a decreasing trend as the processing process progressed, while the intensity of the mellowness, fresh and brisk taste, and aroma showed an increasing trend. TOPSIS analysis showed (Figure 4C) that withering and fermentation had the greatest effect on Rougui taste characteristics in terms of bitterness and astringency, followed by mellowness.

Withering and fermentation are key steps in tea processing, and different teas show different changes in their taste characteristics after undergoing withering and fermentation [38]. For example, after the withering of green tea, the content of polyphenols and flavonoids decreases significantly, and the intensity of the bitterness and astringency of the tea decreases significantly [39]. After the withering and fermentation of black tea, the intensity of the bitterness and astringency of the tea significantly decreases, while the intensity of the sweetness and mellowness significantly increases [10]. The fermentation of Fu brick tea results in a significant decrease in astringency, bitterness, and sourness intensities and a significant increase in mellow intensity [16]. The intensity of bitterness and astringency of Liupao tea significantly increases after withering and fermentation [40]. It is evident that significant differences existed in the impact that withering and fermentation had on the taste characteristics of different teas. Rougui, the subject of this study, belongs to oolong tea, and withering and fermentation were conducive to increasing the intensity of the mellowness, fresh and brisk taste, and aroma while decreasing the intensity of the sweetness, bitterness, and astringency, and withering and fermentation were the most important in significantly reducing the intensity of bitterness and astringency.

## 4. Conclusions

**Figure 5:**
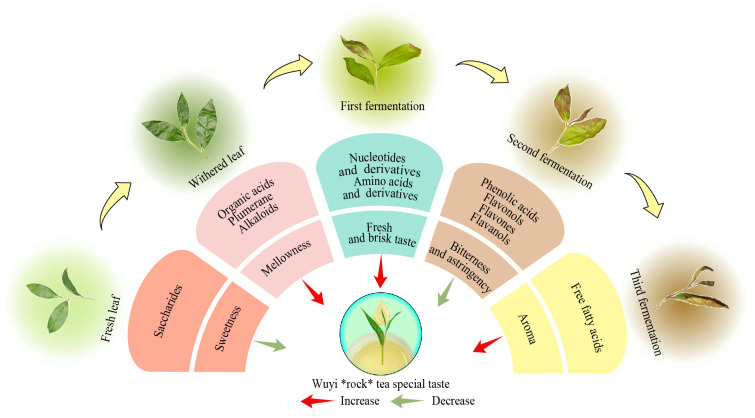
Effect of primary processing on tea quality formation.

In the present study, the effects of withering and fermentation on the metabolites and taste characteristics of Rougui were analyzed, and the results revealed (Figure 5) that the metabolite content in tea leaves significantly increased upon processing. Withering and fermentation promote the transformation and accumulation of metabolites in tea leaves and increase the level of metabolites. The content of 40 key metabolites significantly changed during Rougui withering and fermentation, and they were categorized into 13 groups based on secondary classification. It was further found that the content of the five groups of metabolites mentioned above, such as saccharides, phenolic acids, flavonols, flavones, and flavanols, showed a significant decreasing trend with processing, while the content of eight groups, such as organic acids, plumerane, alkaloids, nucleotides and derivatives, amino acids and derivatives, lysophosphatidyl choline, phenolamine, and free fatty acids, showed a significant increase. After withering and fermentation, the key metabolites that changed significantly in Rougui showed five main taste characteristics, namely mellowness, sweetness, fresh and brisk taste, bitterness and astringency, and aroma. For Rougui, withering and fermentation were conducive to increasing the intensity of the mellowness, fresh and brisk taste, and aroma while decreasing the intensity of the sweetness and bitterness and astringency, and withering and fermentation were the most important to significantly reduce the intensity of bitterness and astringency. It is evident that withering and fermentation are pivotal processes in the development of the unique taste characteristics of Wuyi rock tea, Rougui. This research provides a valuable reference for refining the Rougui processing technology and crafting a unique flavor.

## Data Availability

The original contributions presented in the study are included in the article/supplementary material, further inquiries can be directed to the corresponding author.

## Supplementary Materials

The following supporting information can be downloaded at: https://www.mdpi.com/article/10.3390/foods13233955/s1, Table S1: UPLC-MS/MS determination data of tea leaf metabolites at different processing stages.
